# Digital Catalysts for Noncommunicable Disease Prevention Serious Games and Gamified Applications: Framework Design Study

**DOI:** 10.2196/69246

**Published:** 2025-08-27

**Authors:** Christoph Aigner, René Baranyi, Thomas Grechenig

**Affiliations:** 1 Research Group for Industrial Software, INSO TU Wien Wien Austria

**Keywords:** serious games, gamification, framework, interoperability, noncommunicable disease prevention

## Abstract

**Background:**

Unhealthy behaviors can cause so-called noncommunicable diseases (NCDs), which are on the rise. Notable examples include chronic respiratory diseases, diabetes, cardiovascular diseases, and various types of cancer. They are responsible for approximately 41 million deaths annually, which accounts for a staggering 74% of all global deaths. Major risk factors include physical inactivity, the use of tobacco, unhealthy diets, the harmful use of alcohol, and poor mental health, which can be classified as modifiable behavioral risk factors. Other factors include metabolic and environmental risk factors, such as air pollution. Many individuals struggle to make informed decisions about their health, which contributes to the risk factors mentioned earlier and, ultimately, can lead to the development of one or more NCD.

**Objective:**

This research presents design and standardization considerations to enable the exchange of medical and game data to maximize their impact and usefulness. Serious games and gamified applications that strategically use behavior change techniques and educational content can help users change their behavior on a lasting basis, thereby reducing the aforementioned NCD risk factors. Still, each of them is currently independently designed and cannot interact with other applications.

**Methods:**

We previously developed serious games and gamified applications to prevent NCDs. These served as the foundation of an interoperable framework for NCD prevention games and applications. On the basis of a comprehensive analysis, 6 key areas were identified, ultimately leading to a framework definition that was then evaluated against the already-developed games and applications.

**Results:**

This paper presented a novel interoperable framework to support the design and development of serious games and gamified applications that enable individuals to achieve sustainable behavior change and improve their overall health and well-being by defining 6 key areas, emphasizing interoperability, and exchanging meaningful medical and game data.

**Conclusions:**

The framework presented in this study covers the major design and implementation aspects of NCD prevention games and applications in 6 key areas. Therefore, researchers should consider these guidelines when creating novel serious games and applications in those areas. The framework also intensively encourages the use of standards in the domain of medical informatics to ensure the semantic interoperability of patients’ data produced. Thus, it promotes the exchange of meaningful data to improve patient care and anonymous data use for research.

## Introduction

### Problem Description

Many people find it hard to make conscious decisions about their health, including their physical and mental well-being. Whether it is about eating healthy food, quitting smoking, reducing alcohol consumption, exercising, or maintaining good mental health, changing one’s behavior for the long term can be challenging. According to Bouton [1], behavior change is difficult because new behavior does not erase the old behavior, and new learnings can be relatively specific to the context in which they were learned. Coorevits and Coenen [2] stated that there is a high attrition rate among consumers who no longer wear their fitness trackers. The study by Alshawmar et al [3] had similar findings regarding fitness apps. They observed that, despite the potential benefits, many downloads, and high use rates of fitness apps, studies indicate a significant dropout rate after a short period of use [3]. Even if someone manages to make substantial changes, they often fail to last. Therapies such as psychotherapy and dietary treatment are promising in helping people achieve lasting behavior change, but they can be expensive and may only sometimes be covered by health insurance. For instance, there is compelling evidence of income-based disparities in access to psychotherapy and other mental health services, as seen in current conditions in Australia and Canada [4].

Noncommunicable diseases (NCDs) are on the rise. They are not contagious but can be caused by unhealthy behaviors [5]. Some major examples include cardiovascular diseases, cancers, diabetes, and chronic respiratory diseases. These diseases are responsible for an estimated 41 million deaths per year, which is about 74% of all deaths globally [6]. Major risk factors include physical inactivity, the use of tobacco, unhealthy diets, the harmful use of alcohol, and poor mental health. These can be classified as modifiable behavioral risk factors. Other factors include metabolic factors, such as increased blood pressure, overweight, hyperglycemia, and hyperlipidemia, and environmental risk factors, such as air pollution. To effectively combat NCDs, it is crucial to reduce these risk factors [6]. In the work of Zhao et al [7], it was shown that while increasing age remains a nonmodifiable risk factor for cancer, and thus for an NCD, the incidence for patients aged <50 years is rising. Early-onset cancer deaths increased by a tremendous 79.1% between 1990 and 2019. According to Zhao et al [7], risk factors identified for early-onset cancers are very similar to the ones specified by the World Health Organization for NCDs.

Zhao et al [7] also projected that the global incidence would increase by 31% in 2030. Their work clearly shows that NCD prevention would be very beneficial, even for younger age groups. Another aspect that was long overlooked is mental health disorders in combination with NCD prevention. O’Neil et al [8] showed that NCD prevention and control have historically focused on cancer, cardiovascular diseases, diabetes, and chronic respiratory diseases. Despite recent developments in the integration of common mental illnesses, the prevention and control of these disorders remain largely separate and independent; therefore, O’Neil et al [8] proposed a shared framework for disease prevention and control. An article on linking mental health and NCD alliance campaign priorities, created for the 2018 United Nations High-Level meeting on the Prevention of NCDs, also proposed that governments should address the common risk factors and systems barriers to reducing premature and preventable distress and death by integrating mental health into the response to NCDs [9]. According to Jacka et al [10], there is evidence that could indicate that unhealthy diets can also be risk factors for mental health disorders, particularly depression and dementia, and therefore can be considered a multiplier for the risk of developing NCDs.

Dörner et al [11] described serious games as digital games designed to entertain and achieve additional goals, known as characterizing goals. These additional goals can be related to learning, training, education, health-related use cases, or other applications. Numerous categories of serious games exist within the digital health domain, including exergames promoting physical activity, medical education games and simulations, lifestyle behavior change games, and medical diagnosis learning games. Serious games can take various forms, such as simulations, role-playing, and educational games [12]. They incorporate textual, graphical, haptic, and audio elements to create immersive gaming experiences [13,14]. It is important to note that serious games are distinguished from profession simulators and virtual reality apps due to their goal-oriented nature [12].

It is also essential to differentiate between a serious game and the terms gamification or gamified application. Gamification uses game elements within nongaming systems to enhance user experience and engagement. This concept involves adding game-like elements to nongaming applications and platforms, a process known as “gamifying.” According to Dörner et al [11], gamified applications are typically, but not necessarily, less like a game than full serious games, as they may only incorporate specific game elements or mechanics. Table 1 presents a short overview, distinguishing the 2 terms.

**Table 1:** Comparison of the terms “serious game” and “gamified application.”

Term	Definition	Key characteristics
Serious game	A game that is designed for a primary purpose other than entertainment; used in settings such as classrooms, workplaces, or health care [12]	Digital game of any particular game genre; categorization according to the characterizing goal [11]
Gamified application	Transfer of game methodologies or elements to nongame applications and processes [11]	Adds game elements to a nongame area; the result of gamification is not necessarily a game [11]

A key way for individuals to lower the risk of NCDs is by modifying their lifestyle. Although people are becoming more aware of medical conditions and risk factors involved with NCDs and therefore are more inclined to change their lifestyles, which can be partially attributed to the impact of mass media, most are still finding it hard to change their behavior and maintain it for an extended period. The so-called behavior change techniques (BCTs) can be used to overcome the problems described earlier. Michie et al [15] define BCTs as structured interventions to modify behavior incorporated as active components in an intervention. For example, the goal-setting BCT can be used to define a daily walking goal or to specify how many fruits or vegetables one should consume per day. Another example of a BCT is the self-monitoring of behavior BCT. With this BCT, the individual could be asked to record, in a personal diary, whether they have engaged in a daily 2-minute toothbrushing routine before bedtime [16]. These BCTs were successfully used in serious games and gamified mobile apps. Baranowski et al [17] concluded that serious game–based behavior change is an exciting form of media-based intervention, and the many desirable outcomes warrant moving forward in this area.

Semantic interoperability can be described as the ability to automatically interpret information that is exchanged meaningfully and accurately to produce useful results as defined by the end users of both systems [18]. Health data produced by games and apps must be defined in an interoperable way. As a solution, standards within the domain of medical informatics, such as Health Level 7 (HL7), Integrating the Healthcare Enterprise (IHE), and openEHR, can be used. Interoperability is hard to achieve because it needs to be ensured that the understanding works horizontally across business processes and also vertically within computer system suppliers and between users and developers who speak different dialects [19]. Oemig and Snelick [20] emphasized that clear standards and adherence to these standards are essential for creating reliable, effective, usable, and interoperable health care information systems, highlighting the crucial role of standards. However, Peters et al [21] stated that patients and their caretakers would benefit considerably from integrating semantically rich and standardized health data into existing hospital information systems or electronic health records (EHRs).

We have already published various serious games and gamified applications for NCD prevention and showed that these can be useful and are effective in behavior change. Furthermore, we have already published a baseline for an interoperable framework, comprising a comparative analysis and the definition of a requirement catalog [22]. Each of the developed application’s results, conclusions, findings, and additional analyzed projects were the foundation for constructing an interoperable application framework for behavior change–driven serious games and gamified mobile applications to combat NCDs presented in this paper. The framework includes best practices for designing and implementing games and applications specific to preventing NCDs. It also intensively encourages the use of standards within the domain of medical informatics to ensure the semantic interoperability of patients’ data produced from the applications and games mentioned earlier.

### Related Work

#### Frameworks for Serious Games and Gamification

Peters et al [21] proposed a framework for collecting health-related and game data from serious games and applications that cover the requirements to interpret data from various health and game sources using a decision support system (DSS)–based approach. The initial motivation for creating the framework was the Integrating Entertainment and Reaction Assessment into Child Cancer Therapy (INTERACCT) project, a serious game for young oncology patients. The game world consists of a 2.5D adventure game where the player has to complete procedurally generated levels filled with hidden treasures and hostile avatars. A huge part of the framework’s design is a DSS that calculates structured health scores based on the medical and game data. Peters et al [21] stated that it was challenging to design a ubiquitous data model for serious games for health, mainly because of the wide variety of possible games, mechanics, and medical topics. This observation shows the importance of well-made implementation guides (IGs) focusing on well-defined medical areas, such as NCD prevention, and using current interoperability standards. The framework suggests using standards, but no IGs or interoperability profiles are provided.

Yusoff et al [23] developed a conceptual framework for serious learning games. It combines learning and pedagogy theory with gaming requirements. The framework includes components such as “capability,” which refers to the cognitive, psychomotor, and affective skills players develop in the serious game; instructional content; intended learning outcomes; game attributes supporting engagement; learning activities, reflection on these outcomes; genre; game mechanics; and achievements, such as scores, resources collected, or time to reach goals.

The eAdventure platform is a tool for creating educational adventure games for various domains, including health sciences. It integrates into Moodle-like platforms using the Sharable Content Object Reference Model (SCORM) but solely focuses on one type of video game [24].

Another example is the work of Stanescu et al [25], which proposes a multidimensional interoperability framework for serious games that integrates 3 key dimensions: the core components, the ecosystem, and external factors. According to them, the research on serious game interoperability should focus on standardization, which means the creation of functionally interchangeable items; interchangeability, which refers to identifying methods that would make game components interchangeable; standards adoption, which involves creating adaptable solutions; open system architecture, which provides a modular design that defines key interfaces; and unique specifications and proprietary devices, which indicate that proprietary devices may be counterproductive but are often necessary to provide the needed functionality. Their work focuses on serious learning games.

Cowan and Kapralos [26] focused on technical insights into serious game development by conducting an extensive literature review that examined frameworks and game engines, including a summary of their features. According to their study, the frameworks Second Life, Unity, Unreal, Flash, XNA Game Studio, and the Torque Game Engine were extensively used because they had the highest search results. They concluded that developers primarily used technical frameworks that were not originally designed for serious games but for commercial entertainment games.

#### Interoperability Within Serious Games and Gamified Applications

Digital health applications, especially mobile apps, can (as of January 2023) export their data in a semantically interoperable way into the German national EHR. The term “DiGA” (digital health applications; German: “Digital Gesundheitsanwendungen”) is a German term [27]. The DiGA Toolkit project, currently released as version 1.1.0, allows storing data in the EHR (Elektronische Patientenakte) using HL7 Fast Healthcare Interoperability Resources (FHIR) as part of the so-called Medizinische Informationsobjekte [27]. According to Mittermaier et al [28], physicians can already prescribe authorized DiGAs to patients, which can be reimbursed by statutory health insurance. They also stated that DiGAs can support the field of internal medicine. Although the DiGA profiles already define useful data structures for digital health applications, such as diary entries, questionnaires, and lifestyle factors, they currently do not focus on segments for serious games or gamification for health.

The German Institute for Standardization Registered Association (DIN; German: Deutsches Institut für Normung eV) published a specification for serious game metadata in 2018. This specification defines a standardized format for developers and publishers to describe their games so that potential users can find them more easily [29].

SCORM is a standard that can be used with learning games. It outlines a method for delivering e-learning content across various platforms. Its key components include the content aggregation model, which establishes a framework for packaging learning content, and the runtime environment, which specifies an interface to facilitate communication between the learning content and the system that initiates it [30].

#### Research Gaps

The framework proposed in this paper defines guidelines for designing and developing games and applications in 6 key areas. It emphasizes semantic interoperability by incorporating state-of-the-art standards from the medical informatics domain. Furthermore, it will predominantly focus on behavior change–driven games and gamified mobile apps to prevent NCDs. Both are novel approaches within serious games and gamification in health. Although rudiment framework approaches already exist, they either focus more on serious games for education or e-learning, such as the SCORM standard, or general serious games for health and try to use DSS approaches without constructing actual IGs for the use within national or personal EHRs and thus not allowing medical and game data to be further used for primary care or secondary use within science.

Although IGs for the interoperable exchange of medical data exist for mobile health apps, such as the DiGA Toolkit in Germany [31], they do not specifically focus on serious health games or gamified health apps. They also do not currently use the latest version of HL7 FHIR, which includes newly added resources relevant to serious game data for NCD prevention.

### Summary

Serious games for health, particularly within NCD prevention, have been shown to be effective; however, solutions are scattered, and standardization is still lacking. Because current frameworks for serious games and gamified health applications within this field notably lack IGs for integrating medical and game data into EHRs, and current mobile health app conventions do not address serious health games or use the latest medical informatics standards for NCD prevention, this study proposes a novel interoperable framework for serious games and gamified applications aimed at promoting sustainable behavior change and enhancing health, thereby preventing NCDs. It outlines best practices for designing and implementing applications, facilitating the structured exchange of data for personal or national EHRs, and supporting clinical research. Built on previously published serious games and gamified applications, the framework began with an analysis and a best-practice catalog, which was shared at the International Conference on Healthcare Service Management in mid-2024 [22]. These findings then served as the foundation for the analysis, design, and development of the technical framework, which is divided into 6 key areas considered relevant for NCD prevention serious games and applications. Our claim is to develop a high standard for this field that can be used as a starting point for others.

## Methods

### Overview

**Figure 1:**
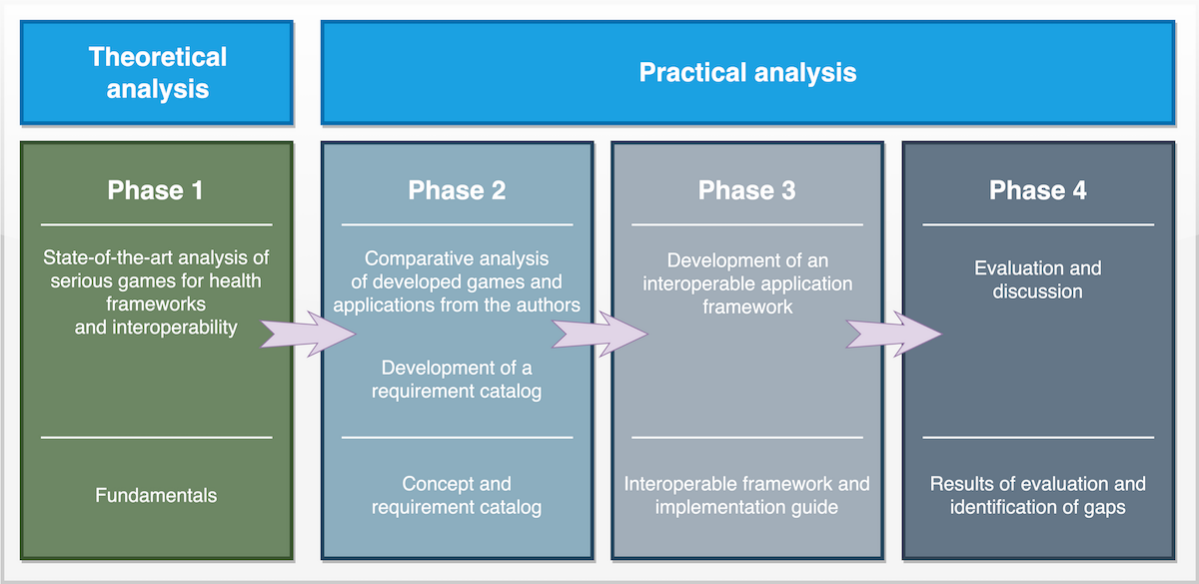
Methodology used in this study.

The methodological approach for this work consisted of 4 principal phases, as shown in Figure 1. The first 2 phases, the fundamental research, including the study of the state of the art, and the analysis of developed games and applications, which led to the definition of a requirement catalog, were partially completed within the initial work that had already been published as part of a conference paper [22]. The Results section refines and summarizes these 2 phases in the context of this particular work. In the third phase, the final interoperable framework was designed and developed based on the findings of the first 2 phases. The results were a collection of best practices and an IG using standards defined by HL7 International. In the final phase, the framework was evaluated against the analyzed games and apps, and gaps were identified.

### Ethical Considerations

This work is based solely on the analysis and synthesis of previously published literature and publicly available information. As such, formal approval from an institutional review board or ethics committee was not required.

## Results

### Overview

This section provides comprehensive insights into designing and developing an interoperable framework for NCD prevention games and applications. The Analysis and Requirement Catalog subsection summarizes the comparative analysis and the definition of the requirement catalog that was made beforehand and has already been published by us. This study was extended with 2 further applications published by some of the authors of this study, MoodBooster, a gamified student mental health app [32], and DiaBeaThis, a gamified self-tracking portal to control the blood glucose level of patients with diabetes [33]. The Definition of the framework subsection describes every aspect of the framework in detail. The framework defines the best practices and design guidelines and provides an IG for the meaningful exchange of medical and game data. The Evaluation subsection evaluates the framework against the analyzed applications and tries to identify gaps.

### Analysis and Requirement Catalog

Table 2 presents an overview of the analyzed applications.

**Table 2:** Summary of the comparative analysis.

ID	Analyzed applications	Type of application	Platform	Main noncommunicable disease risk factor	Secondary (clinical) factors	Studies
G1	BreathIn	Serious game	Android	Smoking	Well-being	Aigner et al [34,35]
G2	Food Pyramid Escape	Serious game	Android	Nutrition	Well-being and nutritional education	Aigner et al [36]
G3	Nutrition Garden	Gamified mobile app	Android	Nutrition	Well-being and nutritional education	Aigner et al [37]
G4	NutritionRush	Serious game	Android	Nutrition	Well-being and nutritional education	Baranyi et al [38]
G5	NutriMine	Serious game (modification)	PC	Nutrition	Well-being and nutritional education	Aigner et al [39]
G6	recoverApp	Gamified mobile app	Android and PC	Mental health	Depression, rehabilitation, injury, and pain	Aigner et al [40]
G7	MoodBooster	Gamified mobile app	Android	Mental health	Well-being and positive psychology	Aigner et al [32]
G8	DiaBeaThis	Gamified web application	Web (PC and mobile)	Hyperglycemia	Well-being	Baranyi et al [33]

The analyzed applications included BreathIn, a serious game for smoking cessation [34,35]; Food Pyramid Escape, a serious learning game for healthier eating [36]; Nutrition Garden, a gamified food tracker [37]; NutritionRush, a serious game for better nutrition [38]; NutriMine, a serious modification for Minecraft, also concerning nutrition [39]; and recoverApp, a gamified app for better mental health for patients in rehabilitation [40]. The newly published gamified app MoodBooster for student mental health [32] and DiaBeaThis, a gamified blood glucose tracking web application published in 2018, relevant to this framework [33], were added to the analysis after the publication of the first part of this work. The 8 selected applications provided illustrative examples and focused especially on nutrition, as overweight and obesity are significant NCD risk factors. According to the World Health Organization [41], 2.5 billion adults aged ≥18 years are overweight, including more than 890 million adults living with obesity, with high prevalence especially in Western countries—67% in the region of the Americas, for example. In addition, the model for the framework is considered an open model that is planned to be continually developed [41].

MoodBooster is a mobile, gamified app that supports good mental health for university students. The prototype runs on the Android (Google LLC) platform. The app tracks mood and stress-related symptoms and includes an educational hub that covers symptoms and the physical and cognitive impact of stress on the human body. The hub also gives students information on how to deal with stress-related symptoms, proper diet, and physical activity. The app also includes features for stress relief, including exercise guides [32]. Furthermore, the app consists of serious minigames. These are Just Breathe! (centered on focused breathing exercises); Draw! (a digitized coloring book to offer relaxation); Wipe Out Worry (a game where the player needs to destroy a castle of predefined negative thoughts); and Mind Your Senses (focusing on the psychological concept of mindfulness). The app includes badges for achieving goals within the minigames and suggested activities. The app produces patient data, mood-tracking data, stress level questionnaire entries, notes, and badges [32].

DiaBeaThis is a web portal accessible via mobile devices or PCs. Upon logging in, users can create diary entries that record their current blood glucose levels and track instances of hypoglycemia and hyperglycemia, carbohydrate intake, insulin injections, and physical activities. Each new diary entry contributes to the accumulation of experience points. These points directly affect the gameplay of the included game, a card game based on War. Two players face each other in multiple rounds and have to present cards. Cards with higher values lead to a win. By gaining experience points, the player levels up and gets access to higher-value cards, thus achieving advantages within the game. DiaBeaThis includes achievements and a leaderboard, which ranks all patients currently registered within the portal. The application records patient data, blood glucose level, amount of carbohydrates consumed, insulin dosage, physical activity duration and description, state of mind via an emoji, and additional notes [33].

A total of 8 applications were analyzed, and the subsequent comparative analysis revealed 14 requirements that should be considered when developing novel gamified apps and serious games for NCD prevention [22].

### Definition of the Framework

#### Overview

**Figure 2:**
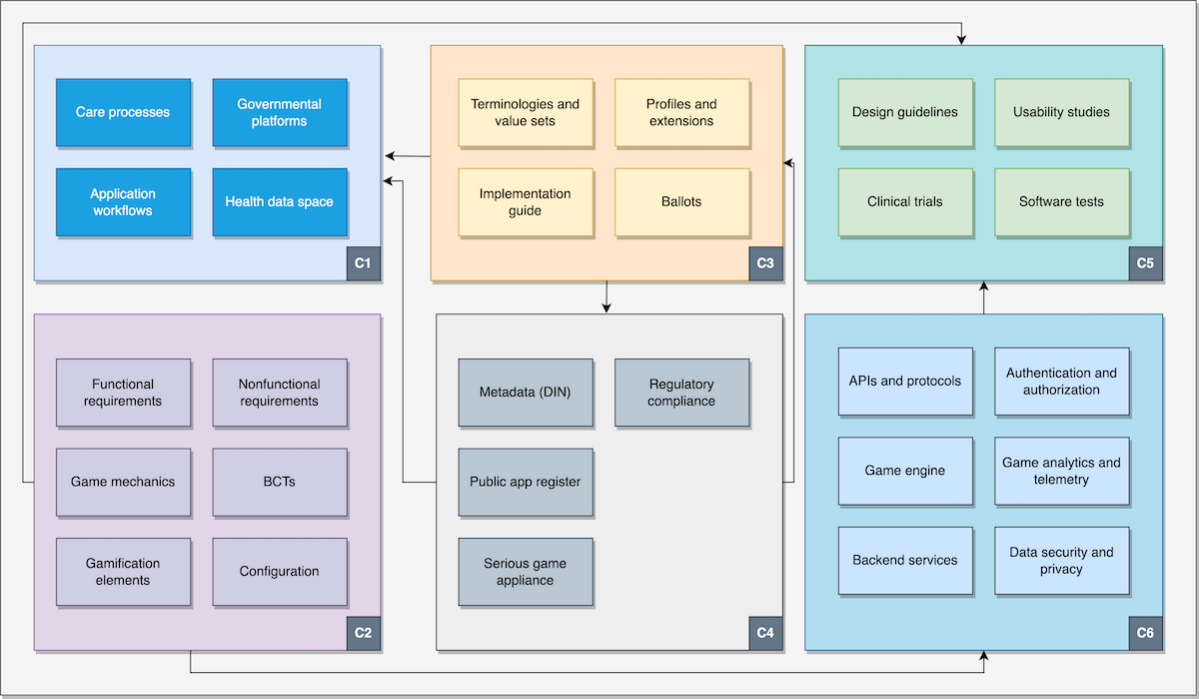
Overview of the defined interoperable framework. API: application programming interface; BCT: behavioral change technique; C1: organizational interoperability component; C2: design and development best practices component; C3: semantic interoperability component; C4: serious games ecosystem component; C5: usability and quality aspects component; C6: technical aspects component; DIN: German Institute for Standardization Registered Association (German: Deutsches Institut für Normung eV); NCD: noncommunicable disease.

The comparison study and the definition of basic requirements were the first steps in a process that combined a series of best practices into a framework focusing on the design, development, ecosystem, and interoperability of NCD prevention games and applications. Figure 2 shows the 6 key areas (organizational interoperability component [C1] to technical aspects component [C6]) of the envisioned framework.

The framework is separated into 6 major, interconnected components. C1 details recommendations for care processes, application workflows, and governmental platforms and considers national and multinational health data spaces. The design and development best practices component (C2) focuses on best practices for designing and developing games and apps by refining the requirement catalog published beforehand, defining sets of functional and nonfunctional requirements and describing the use of game mechanics, gamification elements, BCTs, and configuration aspects. These best practices strongly connect to usability and quality aspects. The third component (semantic interoperability component) focuses on semantic interoperability and, thus, the interoperable definition and exchange of clinical and game data. It includes the definition of value sets and the meaningful use of medical terminologies, defines technical profiles and extensions, and suggests a basic ballot process for the participation of serious games and application developers. The serious games ecosystem component describes practical guidelines for the target group to find games and applications and use them easily. It includes best practices for using metadata, public app registers, and serious game appliances and also tries to give basic suggestions on regulatory compliance. The usability and quality aspects component (C5) covers usability and quality aspects by defining basic design guidelines and offering recommendations for executing usability and clinical studies. The final component (C6) covers technical aspects of serious game development, including application programming interfaces (APIs), game engine use, authentication and authorization, game analytics and telemetry, backend services, and data security and privacy.

#### Organizational Interoperability

##### Care Processes and Application Workflows

Games and apps must be integrated into the care process to support patient behavior change effectively. Health care workers need information about the availability of applications, their intended use, and guidelines for how patients can use them. Therapists and clinicians should be able to prescribe games and applications to patients who could benefit from them. For example, in Germany, applications can already be prescribed as so-called DiGAs [42]. This possibility should be extended to serious games for health. In addition, other countries must adapt so that more patients can benefit from similar opportunities. Patient care plans should determine at which stage of the treatment the application should be prescribed.

Application workflows are very important. BreathIn, for example, should be used as part of an ongoing smoking cessation therapy rather than as a stand-alone intervention. Food Pyramid Escape, by contrast, was designed as a learning app that can be used anytime to teach patients better nutrition and encourage them to change their eating behavior. NCD prevention games should always come with detailed instructions for clinical personnel so that applications can be perfectly integrated into the clinical daily routine.

Therefore, as part of the best practices defined within this framework, developers are strongly encouraged to collaborate with clinicians to define instructions, processes, and workflows during and after development. Because this framework promotes semantically interoperable data extraction into public and personal health records, it is also important to make actual use of the data after patients have finished playing a game or using an application. These data can then be used within future steps in patients’ care plans.

##### Governmental Platforms

Governments should develop platforms to make NCD prevention games and apps available to a wide audience and integrate them into national health promotion and prevention campaigns. This will contribute to NCD prevention and reduce the risk of developing NCDs.

##### Health Data Space

Health data space initiatives should include interoperable NCD prevention games and applications to promote the exchange of medical data across national borders. This gives patients greater control over their data and facilitates secondary use of clinical data for research, innovation, and policy making [43]. An example is the European Union (EU) initiative European Health Data Space (EHDS), which is currently being developed [44]. The EHDS regulation aims to establish a common framework for the use and exchange of electronic health data across the EU [44]. EHDS enables researchers to access large-scale health data through a clear and structured system that facilitates the identification of available data, its location, and its quality, ultimately leading to more cost-effective access to high-quality health information [44]. In the context of NCD prevention games and applications, this can include all health and game-related data elements outlined in the IG in an anonymized and secure manner. In addition, policy makers and regulators can benefit from easier, more transparent, and cost-efficient access to electronic health data for public health monitoring, enhanced health care system efficiency, and assured patient safety.

This framework proposed integrating NCD prevention games and applications into such health data spaces, especially because it encourages data exchange in a semantically interoperable way.

##### Summary

Within this key area, care processes; application workflows; and the integration of government platforms and health data initiatives, such as the EHDS, were explored, as they are essential for the adoption and effectiveness of NCD prevention applications in real-world clinical settings.

#### Design and Development Best Practices

##### Requirement Catalog

A requirement catalog for NCD prevention games has already been published as part of the preliminary work on this framework [22]. The catalog was enhanced for this framework’s definition by adding the fulfillment level and categorizing the requirements into functional and nonfunctional, as presented in Table 3.

**Table 3:** Requirement catalog [22].

ID	Description	Requirement level	Requirement type
R01	Integration of behavior change techniques as core game mechanics	Must	Functional requirement
R02	Integration of engaging gamification elements for applications	Must	Functional requirement
R03	Learning and educational aspects	Should	Functional requirement
R04	Emphasizing good interaction design and usability	Should	Nonfunctional requirement
R05	Use of appealing game graphics	May	Nonfunctional requirement
R06	Integration of music and sound effects	May	Nonfunctional requirement
R07	Considering suitable game genres	May	Nonfunctional requirement
R08	Applying intuitive controls	Must	Nonfunctional requirement
R09	Integration of social support and interaction	May	Functional requirement
R10	Availability of games and applications	Must	Nonfunctional requirement
R11	Games and applications should come at a low cost for users	Should	Nonfunctional requirement
R12	Suitable (hardware) platform for low entry barrier	Should	Nonfunctional requirement
R13	Inclusion of therapists and clinicians	Must	Nonfunctional requirement
R14	Ability to extract data in a semantically interoperable way	Must	Functional requirement

For the level of fulfillment, the definition from Request for Comments 2119 was used [45]. “must” means that the definition is an absolute requirement of the specification; “should” means that a requirement should be implemented, but there may be valid reasons in particular circumstances to ignore it; and “may” means the requirement is truly optional. Additional definitions not used within this requirement catalog include “must not” and “should not,” which can be understood as the opposite of “must” and “should” [45].

The specific requirement levels chosen for the catalog were self-determined, based on state-of-the-art research and the evaluations of the 8 analyzed games and applications.

##### Game Mechanics and Gamification Elements

Four projects included in the comparative analysis were applications with gamification elements rather than full-serious games. These applications primarily offer nongaming features, such as a nutrition tracker in Nutrition Garden, blood sugar level tracking in DiaBeaThis, mood tracking in recoverApp and MoodBooster, and gamification elements. Features such as badges, achievements, challenges, and rewards were used successfully and should be implemented by novel gamified applications.

Some game mechanics are more effective for NCD prevention games than others. For instance, BreathIn and Nutrition Rush use a traditional side-scrolling platformer as their main gameplay. This approach is straightforward to grasp, as it draws from concepts popularized in the early days of video gaming during the 1970s and 1980s. While touch controls on mobile devices function adequately, attention should be given to them, as confusing controls could detract from the gaming experience. Food Pyramid Escape presents an adventure-like game where players directly navigate the main character with an on-screen directional pad. It also uses an inventory and an encyclopedia. NutriMine, a modification for Minecraft, was the only first-person game in the analysis. It was designed to work with the Java version of Minecraft for PC, making it easier to control than the mobile version using a mouse and a keyboard.

Overall, platformer and adventure-type games are best suited for serious NCD prevention games because they are easily accessible and can be controlled intuitively, even on small smartphone screens. In particular, 3D and first-person games may be more challenging to access and control on mobile devices, so they are not encouraged for NCD prevention games and applications.

##### BCT Guidelines

Serious games can effectively use BCTs to help with actual behavioral changes in their users. The BCT categories used within this framework came from the BCT Taxonomy (version 1), which describes 93 hierarchically clustered techniques [16]. For example, the BCT “information about health consequences” was implemented by all analyzed apps except recoverApp and DiaBeaThis, whether it was information about good nutrition, the hazards of smoking, or the various effects of bad mental health and how to achieve good mental health. Other important BCTs used by all games except Food Pyramid Escape and Nutrition Rush were the “self-monitoring of behavior” and “feedback on behavior” BCTs. They were mostly used for tracking cigarette consumption, food diaries, calorie tracking, blood sugar levels, and mood and stress tracking.

MoodBooster, recoverApp, Nutrition Garden, DiaBeaThis, and BreathIn used the “incentive (outcome)” BCT. It was mostly used in the form of achievements, badges, and (skill) points. In addition, recoverApp used the BCT “social support” by providing a chat feature and the BCT “graded tasks” by implementing the task feature, allowing the therapist to enter therapy-related tasks for their patients to perform. DiaBeaThis uses the BCT “social comparison” because its included game “War” requires 2 players facing each other.

Overall, the BCTs “information about health consequences,” “self-monitoring of behavior,” and “feedback on behavior” should be implemented in novel games and applications and are therefore recommended as part of this framework, as they were successfully implemented and have been shown to be useful. The “incentive (outcome)” BCT is a good addition and, depending on the game, should be strongly considered when designing new NCD prevention games and applications. Social BCTs such as “social support” and “social comparison” can be successful, but they strongly depend on the app’s target group.

##### Configuration

Configuring certain aspects of serious games and gamified applications can be very beneficial and is therefore encouraged by this framework. This includes setting certain game or level settings, such as speed, time, the player’s lives, achievements, and overall difficulty [46], as well as the general possibility of turning certain features on and off. Configuration options can be important regarding social features because some players can be negatively affected by them, especially if they involve social comparison, for example, a leaderboard, high scores, and badges and achievements, as was shown.

##### Summary

The previous subsections (in the Design and Development Best Practices section) explored the best practices for the design and development of NCD prevention games and applications, especially focusing on key requirements that need to be included when designing novel applications, the integration of game mechanics and gamification elements, how to effectively integrate BCTs, and aspects of configurational options that allow games and applications to adapt to each distinct user.

#### Semantic Interoperability

##### Overview

The ability to exchange data from NCD prevention games and applications meaningfully is one of this framework’s key aspects. Using the data within public EHRs and personal health records can be very beneficial for patients to archive and record their data and help with ongoing treatment and therapy; therefore, it will be helpful for clinicians as well. The data can also be used anonymously for scientific purposes as part of secondary use. This section describes the developed IG that defines a set of profiles, extensions, and value sets using HL7 FHIR, a standard for exchanging electronic health care data created by the HL7 International organization [19]. The defined profiles encourage the use of current medical terminologies, such as Systematized Nomenclature of Medicine Clinical Terms Clinical Terms for general clinical terms [47] and Logical Observation Identifiers Names and Codes [48] for medical laboratory observations. HL7 FHIR was particularly chosen because of its wide adoption in the United States and Europe as well as its strong focus on implementers and web technology.

##### IG Structure

The developed IG, a collection of profiles, extensions, value sets, and code systems, defines the structure of documents, which are the units of exchange. It represents all relevant data from a specific serious game and gamified application for NCD prevention using HL7 FHIR. Extensions can be used for additional data elements not defined in the standard. Value sets represent actual elements from one or more code systems.

An “FHIR Document” is an immutable bundle with an attested narrative that serves as the unit of data that is exchanged between systems and persisted in health records [49], using integration profiles such as Cross-Enterprise Document Sharing (XDSb) defined in the IT infrastructure technical framework by IHE [50]. XDSb is widely used, for example, within Elektronische Gesundheitsakte, the Austrian national health record, and Elektronische Patientenakte, the national health record in Germany, and provides a standards-based specification for the registration, distribution, and access of medical records [51]. It aims to offer a standards-based specification for sharing documents across various health care organizations, from private practices to large clinics. The XDSb profile presumes that each organization is part of one or more XDSb Affinity Domains—groups of health care entities that have mutually agreed to collaborate based on shared policies and infrastructure [51].

Technically, an FHIR document is an FHIR resource of type “bundle” that starts with an FHIR “composition” resource and contains various other resources of a set version that are referenced within that “composition.” The HL7 FHIR standard defines a large set of resources that can be used for various clinical and administrative use cases. To further customize them, HL7 FHIR defines profiles that specify acceptable codes, contain new elements in the form of extensions, and specify constraints on existing elements and data types [52].

The collections of profiles, extensions, value sets, and code systems were designed using FHIR Shorthand (FSH). Shorthand is a domain-specific language for defining FHIR profiles. The first full version was released in March 2020. Many FHIR community projects, including the National Health Record Initiative in Austria (Elektronische Gesundheitsakte) [53], adopted FSH.

The transpiler “SUSHI” was used to process the defined FSH files and transform them into FHIR artifacts [54]. The HL7 FHIR IG Publisher tool was used to create a standardized FHIR IG. This exact combination of tools was chosen because the alternative would have been using complex Microsoft Excel spreadsheets or a graphical tool called “Forge,” which is only free of charge for single users and only available for Microsoft Windows [52]. FSH has many advantages. It is concise, readable, and understandable, and changes can be made via text operations; because it is text based, it is suited for source code version control, has error checking, and incorporates best practices [52]. The basic requirement for an FSH-SUSHI setup is a recent computer with at least Java 17 and Node.js 18 installed.

Furthermore, the FHIR IG Publisher, written in Java, uses the Jekyll framework to render complete IGs as web pages. The actual shorthand files were created using Visual Studio Code (Microsoft Corporation) because it offered very useful plugins for HL7 FHIR, such as FHIR Tools [55] and FSH language support [56]. Version 5.0.0 of FHIR was used, which included new resources that were of particular interest for this IG, such as the NutritionIntake and NutritionProduct resources.

Each FSH file has the same structure. The first block contains the definition of the profile. The second block defines the parent resource, the title, and the resource description. Finally, the third block represents all the rules the profile should include. Elements can be marked as ‘must support’ or limited in their cardinality. The profile can also restrict references to specific other profiles. For example, within the NCD patient resource, the reference to the attending physician was limited to the “NCD Practitioner” profile.

**Figure 3:**
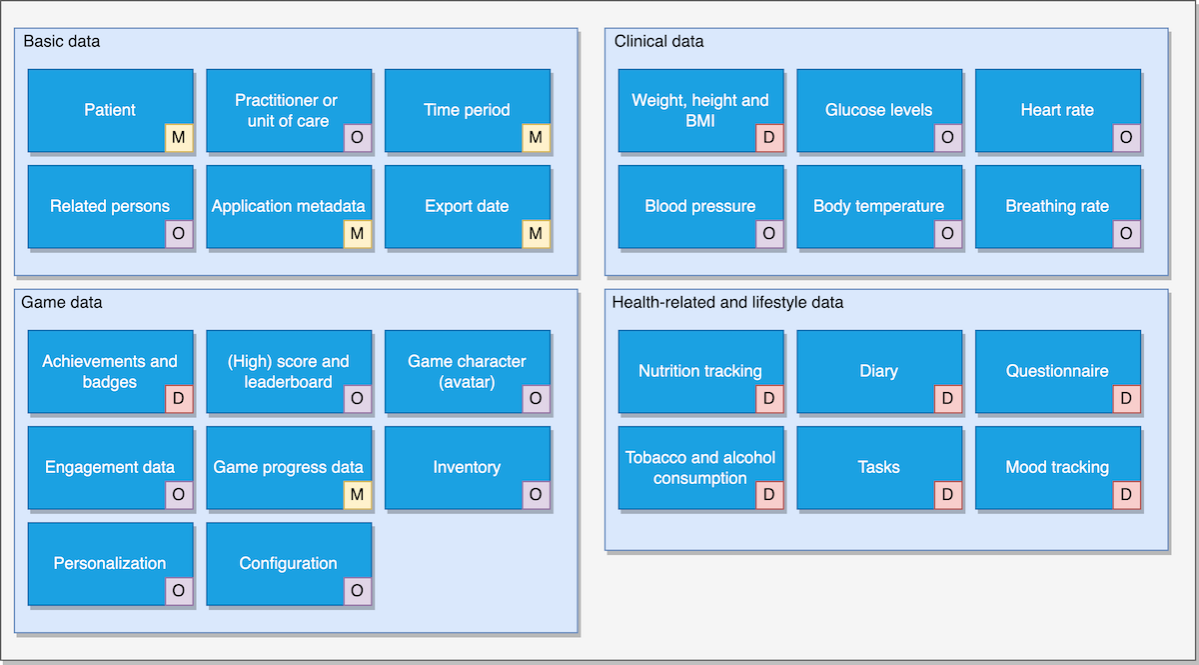
Data model of the implementation guide. D: dependent and mandatory element for particular games or applications; M: mandatory element; O: optional element; SG: serious game.

Figure 3 gives an overview of the defined data model. All relevant data elements together form the FHIR document, which is technically represented as an FHIR Bundle resource. Each component was translated into an HL7 FHIR profile using FSH.

The main element is the “NCD Bundle,” which contains the “NCD Composition” as its first element. The “NCD Composition” comprises 3 extensions: the time period of the exported data, the export date, and a free text field for metadata containing the DIN-specified metadata for the game (refer to the Serious Games Ecosystem section). The profile also includes slices for linking the practitioner and related persons. It contains 3 compositions that comprise clinical data, game data, and health-related and lifestyle data, as depicted in Figure 3. Two code systems, along with their value sets were created: “NCD Gamedata,” which codes the different types of game data (achievements, high scores, and others), and “NCD Lifestyle data,” which encodes the various types of lifestyle data (mood, alcohol consumption, tobacco use, and diary entry).

The IG Publisher rendered all profiles, code systems, and value sets as a web page. The web page includes an artifact summary page that overviews all defined resource profiles, extensions, value sets, and code systems. All artifacts can be exported as comma-separated values, Microsoft Excel spreadsheets, or Schematron files.

The IG is considered a ‘draft standard for trial use’ and has not yet been validated in real-world scenarios. For broader industry adoption, it must be validated by national HL7 technical committees and experts. This can be easily achieved because the IG was created with standardized and widely adopted HL7 tools and is publicly available.

##### Ballot Process

If changes to the NCD prevention IG need to be made, all stakeholders must be involved, especially serious game and EHR implementers. They should be able to give feedback and comment on proposed changes before publication. Therefore, the framework suggests implementing and following the HL7 balloting process, which is defined in a previous study [57] and is articulated. First, the stakeholders must sign up to participate in the ballot process. Then, the stakeholders review the proposed specification, provide feedback as ballot comments, and mark their comments as “negative” or “affirmative.” After that, all comments are reviewed, and a decision is made about which changes to the specification will be incorporated. Stakeholders have the opportunity to “retract” or “withdraw” their “negative” comments. Agreed-upon changes to the specification will then be applied. Afterward, a final determination is made as to whether the specification has passed the ballot or needs additional changes. Finally, a recirculation ballot might be necessary in some cases.

##### Summary

The previous subsections outlined an IG for the interoperable exchange of medical and game data using the widely adopted HL7 FHIR standard. Change management was also addressed by defining a ballot process that follows the HL7 standard procedure.

#### Serious Games Ecosystem

##### Metadata (DIN)

Every NCD prevention game or gamified application should incorporate a metadata definition following the DIN standard established by Göbel et al [29]. The metadata is part of the FHIR document (refer to the IG Structure section). These data help categorize applications and make them easier to find. Potential users can also check the suitability for their needs beforehand. The DIN norm defines 3 levels of descriptive elements. In this case, level 1 (core) is the minimum that needs to be supported. The preferred option would be compliance with levels 1 and 2, which contain supplementary descriptive elements. Additional application profiles (level 3) are not required and therefore optional.

##### Public Register

Clinical personnel must be able to find appropriate applications that they can recommend or prescribe to patients (refer to the Care Process and Application Workflow section). This is only possible if public registers of accessible games and applications are available. Therefore, the framework proposes providing public registers that health care professionals can use to search for particular games and applications. Each application must provide specific metadata so that it can be easily found (refer to the Metadata [DIN]) section). Nonprofit organizations, governmental entities, or international entities are encouraged to host such registers.

##### Serious Games Appliance

Serious game appliances can incorporate NCD prevention games and applications. These are specific technical environments for serious games that can run on PCs, smartphones, or specialized hardware. They feature dedicated launchers to streamline game access and overall usability.

##### Regulatory Compliance

Approval for NCD prevention games to be used in clinical settings may be necessary. For example, digital health apps in Germany can be licensed as DiGAs [58], which requires a fast-track procedure according to section 139e of the German Fifth Book of the Social Code [42]. These applications must not currently be used for primary prevention and must be recognized as medical products. This framework encourages developers to undergo certification processes for games and applications in specific health care scenarios. Digital health application programs such as DiGAs in Germany should also include games and applications for primary prevention in the future.

##### Summary

This key area focused on creating a serious games ecosystem that enables building high-quality games and applications by considering a DIN norm centered on metadata, public registers, and applications where games can be found and run, along with regulatory and certification aspects.

#### Usability and Quality Aspects

##### Design Guidelines and Usability Studies

Design guidelines should be followed to provide consistent user experiences. Android and Apple publish official guidelines for mobile apps that are recommended to follow. For web applications, World Wide Web Consortium guidelines should be used. Specific design guidelines provided by game engines or technical frameworks should be followed for games, in general.

##### Clinical Trials

Clinical trials are needed to fulfill certain certification requirements and provide valuable insights into the effectiveness of games and applications beyond smaller evaluations within respective target groups. Therefore, it is highly recommended that NCD prevention application developers participate in clinical trials.

##### Software Testing

Rigorous software testing methods are highly endorsed as part of this framework’s best practices. NCD prevention games and applications can only be effective if they run well without noticeable errors and have concise user experiences.

##### Summary

The previous subsections outlined important software quality aspects by defining design guidelines, rigorous software testing, usability studies, and the meaningful use of clinical trials.

#### Technical Aspects

##### APIs, Protocols, Authentication, and Authorization

HL7 FHIR specifies not only how the various data elements are structured but also how they should be exchanged using an API. The IG, which was developed, also includes a capability statement that specifies exactly how to interact with the FHIR API. It consists of a specification in the “OpenAPI” standard [59] that developers can directly use.

However, if games use proprietary backend systems, this should also be allowed and will not be further specified by the IG. Communication should still be based on common standards, such as representational state transfer web services, and adhere to state-of-the-art security and privacy practices.

##### Game Engine

The analyzed games BreathIn and Food Pyramid Escape use the cross-platform game engine Unity [60]. NutritionGarden, NutritionRush, recoverApp, and Moodbooster were developed as native Android apps. The minigames in Moodbooster were developed with JavaScript. NutriMine, a Minecraft mod, was created with MinecraftForge. DiaBeaThis and the therapist application for recoverApp were implemented as web applications. Various engines and frameworks are available to develop NCD prevention games and applications. Native frameworks for mobile apps or cross-platform game engines such as Unity have been successfully used by analyzed games and applications and are recommended.

##### Game Analytics and Telemetry

Telemetry data can help developers improve games by allowing them to analyze user interactions. Therefore, this framework encourages the collection of telemetry data, provided data security and privacy aspects are considered.

##### Backend Services

Games and applications can use their proprietary backend systems if required for specific use cases. Because all analyzed applications did not use backend services, this framework neither encourages nor discourages the use of such systems. However, developers should adhere to the data security and privacy standards discussed within the framework.

##### Security and Privacy Aspects

Because sensitive data can be stored in proprietary backend systems, games and applications must adhere to common data privacy laws. This framework highly recommends integrating the General Data Protection Regulation for applications and games distributed in the EU [61]. The respective data protection laws must be followed if games and applications are provided in other countries.

Data should always be transmitted in an encrypted manner and, if possible, stored in an end-to-end encrypted form on any backend systems. Encryption ensures that only users have direct access to the data stored on a server on their behalf. Data transmitted to national or personal health records must be encrypted with transport-layer encryption. Data storage depends on the specific health record system’s technical specifications and is not within this framework’s scope.

##### Summary

The final key area focused on technical aspects, such as APIs, protocols, the use of game engines, backend services, and security and privacy concerns, which are especially important for personal and medical data.

### Evaluation

This section compares the framework’s 6 key areas, C1 to C6, with the actual state of the games and applications that were developed beforehand and served as the foundation for defining the best practices. Table 4 presents an overview of the degree of fulfillment in each key area.

**Table 4:** Comparison of the applications in terms of framework key areas and fulfillment (none or partial).

ID	Project	C1^a^	C2^b^	C3^c^	C4^d^	C5^e^	C6^f^
G1	BreathIn	None	Partial	None	None	Partial	Partial
G2	Food Pyramid Escape	None	Partial	None	None	Partial	Partial
G3	Nutrition Garden	None	Partial	None	None	Partial	Partial
G4	NutritionRush	None	Partial	None	None	Partial	Partial
G5	NutriMine	None	Partial	None	Partial	Partial	Partial
G6	recoverApp	None	Partial	None	None	Partial	Partial
G7	MoodBooster	None	Partial	None	None	Partial	Partial
G8	DiaBeaThis	None	Partial	None	None	Partial	Partial

^a^C1: organizational interoperability component.

^b^C2: design and development best practices component.

^c^C3: semantic interoperability component.

^d^C4: serious games ecosystem component.

^e^C5: usability and quality aspects component.

^f^C6: technical aspects component.

None of the analyzed games and applications currently fulfill the requirements regarding organizational and semantic interoperability (key areas C1 and semantic interoperability component) because they were only being evaluated in smaller clinical settings and are not yet widely available. Furthermore, the defined HL7 FHIR IG was unavailable when these applications were designed and implemented.

All analyzed applications fulfilled at least some of the defined best practices (key area C2) because they successfully implemented BCTs and included engaging gamification aspects and game mechanics.

Except for NutriMine, which was already successfully integrated into a prototypically implemented serious game appliance, the other applications still need to fulfill parts of the key area serious games ecosystem component.

All analyzed applications at least partially fulfilled the key areas of usability and technical aspects (areas C5 and C6). They were also playtested and evaluated with users from the target groups. However, as all analyzed applications are still at a prototypical stage, best practices regarding game engines, APIs, backend services, and security measures have yet to be fulfilled.

Overall, a comprehensive evaluation of all 8 applications revealed that none of them fully fulfilled any of the 6 key areas; some partially fulfilled certain areas, especially key areas C2, C5, and C6.

## Discussion

### Principal Findings

An interoperable framework was developed based on a comparative analysis of 8 serious games and gamified applications for NCD prevention published by or in part by the authors of this paper. The first part, the comparative analysis and basic requirements definition, was done beforehand and published at the International Conference on Healthcare Service Management in mid-2024 [22]. Six games were initially part of that analysis, but 2 games were added for this particular work. The defined requirements were refined and categorized. Table 5 compares the developed framework with work from the state of the art.

**Table 5:** Comparison of serious game (SG) frameworks.

Feature	The framework by Peters et al [21]	The framework by Yusoff et al [23]	The framework by Stanescu et al [25]	Proposed framework
Scope	Collection of health-related data from SGs with a DSS^a^ approach	Conceptual model for serious games, including learning and pedagogy	Multidimensional interoperability framework	Interoperable technical framework for SGs and gamified applications
Target domain	SGs for health	Educational SGs	Educational and general SGs	NCD^b^ prevention SGs and gamified applications
Interoperability	Recommends use of standards but lacks implementation guides	Conceptual only; does not specify interoperability mechanisms	Leverages interoperability between game components, SGs, and learning systems using SCORM^c^ and LOM^d^	Full semantic interoperability
Implementation guidance	Moderate; focuses on the data model, SDK^e^, and DSS	Low; mainly conceptual with few practical details	Medium; includes technical architecture suggestions	High; includes FHIR^f^ profiles, implementation guides, and API^g^ integration examples
Adaptability and extensibility	Inconclusive	Inconclusive	High; multiple technologies supported	Very high; modular FHIR-based resources allow for easy extension and ballot process for changes

^a^DSS: decision support system.

^b^NCD: noncommunicable disease.

^c^SCORM: Sharable Content Object Reference Model.

^d^LOM: learning object metadata.

^e^SDK: Software development kit.

^f^FHIR: Fast Healthcare Interoperability Resources.

^g^API: application programming interface.

The key finding of this work is the definition of an interoperable framework for NCD prevention that consists of 6 key areas. A central part of the framework is the definition of an IG for the interoperable extraction and exchange of medical and game data into EHRs based on the state-of-the-art medical informatics standards HL7 FHIR and FSH for the profile definition. The guide is available through technical artifacts (HTML representation, JSON, Microsoft Excel, and CSV). Moreover, it includes a capability statement with API definitions that implementers can use when designing novel, serious games. In practice, this enables serious games and gamified apps that follow the defined framework guidelines to bundle their collected data into HL7 FHIR documents, which can be represented as either an XML or JSON file, and store it on FHIR-enabled EHRs. This can be either an FHIR facade that receives FHIR documents over a representational state transfer interface and transforms them into the native format of the EHR, or an IHE- or XDSb-enabled EHR web service that allows for cross-enterprise document sharing.

Furthermore, the framework suggests best practices for designing and developing NCD prevention games and applications by providing key requirements and guidelines for BCTs, gamification elements, game mechanics, and general usability and technical aspects. It also addresses organizational aspects such as care processes, governmental platforms, health data spaces, and regulatory compliance.

The framework presented in this study can be a fundamental guideline for building new games and applications to prevent NCDs.

### Comparison With Prior Work

Unlike the first study [22], which included only 6 games and applications in its comparative analysis, this work included 2 more. Furthermore, previous work presented only a catalog of requirements. This work presents a complete, interoperable framework comprising 6 key areas.

### Future Work

The next steps of this work will involve refining the framework by including more applications and focusing on tools, documentation, reference implementations, and partnerships as well as using it in practice by storing and extracting actual data from EHRs. In addition, the use of large language models for HL7 FHIR data in NCD prevention is being evaluated because this field is emerging in semantic interoperability research [62].

### Recommendations

This study’s findings, based on the games and applications built beforehand, are valuable for the crucial area of NCD prevention. The use of semantically interoperable data formats presents an opportunity to enhance not only primary care for patients but also enables the use of data for scientific and regulatory purposes as part of secondary use, particularly within supranational health care projects such as the EHDS. This is especially relevant given the rising use of large language models and artificial intelligence.

### Limitations

Although we consider the framework’s findings very valuable for the crucial area of NCD prevention, some limitations should be considered.

First, the most significant limitation arises from the number of applications analyzed. A total of 8 applications were evaluated. Most of the games and applications were created by us to understand the mechanisms and users involved, but additional applications were also used, and still they do not represent a full set of available applications and all categories. Future work should include more applications to refine the constructed interoperable framework further. As a result, the best practices established regarding the design and development of NCD prevention games and applications within the framework can be considered limited due to the narrow sample size. A more comprehensive approach that incorporates a broader range of NCD prevention games and applications could create a more effective interoperable framework for NCD prevention that does not inadvertently reflect the specific characteristics of the selected games and applications, potentially overlooking other successful strategies or innovations available in different applications. Furthermore, the evaluation was limited in depth. Therefore, future work should also focus on a more comprehensive assessment with pilot implementations, usability studies, and stakeholder interviews.

Second, the technical IG, developed as part of the interoperable framework, which uses HL7 FHIR and FSH to define extensions and profiles for NCD prevention games and clinical data, can be viewed as a prototype and will be used in practice as part of future work as well. For instance, some elements are currently represented as string values and must be accurately parsed by consuming systems. For example, the DIN metadata should be defined as an FHIR element instead of a string value.

Third, because the framework has a strong focus on technical and engineering aspects, developers require a specific background and knowledge of HL7 FHIR and other relevant technologies. To address this limitation, future work should focus on providing user-friendly tools and documentation as well as developing modular and open-source components, such as reference implementations, and establishing partnerships with academic institutions and national or local health networks.

### Conclusions

This research was based on an investigation of the state of the art and a comparative analysis of 8 serious games and gamified applications. This led to the design and development of an interoperable framework for NCD prevention in 6 key areas. The framework defined a novel approach to best practices for developing serious NCD prevention games and gamified applications that should be considered when developing new projects in that area. This framework was then evaluated against the 8 games and applications mentioned earlier, and gaps were identified.

## Abbreviations

API: application programming interface
BCT: behavior change technique
C1: organizational interoperability component
C2: design and development best practices component
C5: usability and quality aspects component
C6: technical aspects component
DiGA: digital health application (German: Digital Gesundheitsanwendungen)
DIN: German Institute for Standardization Registered Association (German: Deutsches Institut für Normung eV)
DSS: decision support system
EHDS: European Health Data Space
EHR: electronic health record
EU: European Union
FHIR: Fast Healthcare Interoperability Resources
FSH: Fast Healthcare Interoperability Resources Shorthand
HL7: Health Level 7
IG: implementation guide
IHE: Integrating the Healthcare Enterprise
NCD: Noncommunicable Disease
SCORM: Sharable Content Object Reference Model
XDSb: Cross-Enterprise Document Sharing

## Data Availability

The datasets generated or analyzed during this study are available on the web [63].

## References

[1] Bouton ME (2014). Why behavior change is difficult to sustain. Prev Med.

[2] Coorevits L, Coenen T (2017). The rise and fall of wearable fitness trackers. Acad Manag Proc.

[3] Alshawmar M, Tulu B, Hall-Phillips A (2022). Influence of personality traits on the continued use of fitness apps. Proceedings of the 55th Hawaii International Conference on System Sciences.

[4] Bartram M, Stewart JM (2019). Income-based inequities in access to psychotherapy and other mental health services in Canada and Australia. Health Policy.

[5] Non-communicable diseases. International Federation of Red Cross and Red Crescent Societies.

[6] (2024). Noncommunicable diseases. World Health Organization.

[7] Zhao J, Xu L, Sun J, Song M, Wang L, Yuan S, Zhu Y, Wan Z, Larsson S, Tsilidis K, Dunlop M, Campbell H, Rudan I, Song P, Theodoratou E, Ding K, Li X (2023). Global trends in incidence, death, burden and risk factors of early-onset cancer from 1990 to 2019. BMJ Oncol.

[8] O'Neil A, Jacka FN, Quirk SE, Cocker F, Taylor CB, Oldenburg B, Berk M (2015). A shared framework for the common mental disorders and non-communicable disease: key considerations for disease prevention and control. BMC Psychiatry.

[9] Eaton J, O’Donnell K, Westerman L, Adshead F (2018). Linking mental health and NCD priorities. Mental Health Innovation Network.

[10] Jacka FN, Sacks G, Berk M, Allender S (2014). Food policies for physical and mental health. BMC Psychiatry.

[11] Dörner R, Göbel S, Effelsberg W, Wiemeyer J (2016). Serious Games: Foundations, Concepts and Practice.

[12] Damaševičius R, Maskeliūnas R, Blažauskas T (2023). Serious games and gamification in healthcare: a meta-review. Information.

[13] Ma M, Oikonomou A, Jain LC (2011). Serious Games and Edutainment Applications.

[14] Gómez B (2017). Differences between e-learning, gamification and serious games. On Serious Games.

[15] Michie S, Johnston M, Carey R, Gellman MD (2020). Behavior change techniques. Encyclopedia of Behavioral Medicine.

[16] Michie S, Richardson M, Johnston M, Abraham C, Francis J, Hardeman W, Eccles MP, Cane J, Wood CE (2013). The behavior change technique taxonomy (v1) of 93 hierarchically clustered techniques: building an international consensus for the reporting of behavior change interventions. Ann Behav Med.

[17] Baranowski T, Buday R, Thompson DI, Baranowski J (2008). Playing for real: video games and stories for health-related behavior change. Am J Prev Med.

[18] ISO 13606-1:2008(en) health informatics — electronic health record communication — part 1: reference model. International Organization for Standardization.

[19] Benson T, Grieve G (2016). Principles of Health Interoperability: SNOMED CT, HL7 and FHIR.

[20] Oemig F, Snelick R (2016). Healthcare Interoperability Standards Compliance Handbook.

[21] Peters K, Kayali F, Silbernagl M, Lawitschka A, Hlavacs H (2017). A proposed framework for the collection of health-related data from serious games and apps. Int J Serious Games.

[22] Aigner C, Baranyi R, Grechenig T (2024). Foundations of an interoperable framework for serious games and gamified mobile apps in non-communicable disease (NCD) prevention. Proceedings of the 2024 7th International Conference on Healthcare Service Management.

[23] Yusoff A, Crowder R, Gilbert L, Wills G (2009). A conceptual framework for serious games. Proceedings of the 9th IEEE International Conference on Advanced Learning Technologies.

[24] Ger PM (2014). eAdventure: serious games, assessment and interoperability. Proceedings of the 2014 International Symposium on Computers in Education.

[25] Stanescu IA, Stepfan A, Kravcik M, Lim T, Bidarra R (2012). Interoperability strategies for serious games development. Proceedings of the 8th International Scientific Conference eLearning and Software for Education.

[26] Cowan B, Kapralos B (2014). A survey of frameworks and game engines for serious game development. Proceedings of the 14th International Conference on Advanced Learning Technologies.

[27] Weber S, Heitmann KU (2021). [Interoperability in healthcare: also prescribed for digital health applications (DiGA)]. Bundesgesundheitsblatt Gesundheitsforschung Gesundheitsschutz.

[28] Mittermaier M, Sina C, Richter JG, Raspe M, Stais P, Vehreschild J, Wolfrum S, Anthes C, Möckel M (2022). [Practical use of digital health applications (DiGA) in internal medicine]. Internist (Berl).

[29] Göbel S, Caserman P, Hansen J, Bruder R, Abels S, Behrmann M, Hauge J, Limpach O, Reichert M, Junge J, Vogt S, Raubold O, Nowarra N, Kruse R, Roller W, Streicher A, Walter T, Wacker M, Flory C, Martin-Niedecken A (2018). DIN SPEC 91380:2018-06 serious games metadata format. DIN Media.

[30] Buendia FB, Hervás AH (2006). Evaluating e-learning platforms through SCORM specifications. Proceedings of the IADIS Virtual Multi Conference on Computer Science and Information Systems 2006.

[31] DiGA Toolkit 1.1.0. Kassenärztlichen Bundesvereinigung (KBV).

[32] Aigner C, Moser M, Wolfsberger OC, Baranyi R, Hohenegger V, Grechenig T (2024). MoodBooster - a gamified app to support stress reduction in university students. Proceedings of the 2024 IEEE International Conference on E-Health Networking, Application & Services.

[33] Baranyi R, Willinger R, Lederer N, Walcher F, Grechenig T (2018). DiaBeaThis — a gamified self-tracking portal to support people suffering from diabetes mellitus to control their blood glucose level. Proceedings of the IEEE 6th International Conference on Serious Games and Applications for Health.

[34] Aigner C, Zeillinger V, Baur K, Baranyi R, Grechenig T (2023). BreathIn – a serious game to support patients with smoking cessation: analysis and design study for a mobile serious game to help patients quit smoking. Proceedings of the 2023 7th International Conference on Medical and Health Informatics.

[35] Aigner C, Baranyi R, Hoelbling D, Baur K, Zeillinger V, Grechenig T (2023). Analysis, implementation, and assessment of a serious game for smoking cessation: investigating design and playtesting outcomes. Proceedings of the 11th International Conference on E-Health and Bioengineering.

[36] Aigner C, Resch EM, El Agrod A, Baranyi R, Grechenig T (2021). Food Pyramid Escape - a serious escape game for the support of nutritional education in Austria and beyond. Proceedings of the IEEE 9th International Conference on Serious Games and Applications for Health.

[37] Aigner C, Hofmann G, Winkler S, Baranyi R, Grechenig T (2023). Nutrition Garden - a gamified mobile app for motivating people to eat specific food to prevent non-communicable diseases. Proceedings of the 2023 7th International Conference on Medical and Health Informatics.

[38] Baranyi R, Steyrer B, Lechner L, Agbektas GN, Lederer N, Grechenig T (2017). NutritionRush - a serious game to support people with the awareness of their nutrition intake. Proceedings of the 5th International Conference on Serious Games and Applications for Health.

[39] Aigner C, Köck K, Baranyi R, Winkler S, Weindl K, Grechenig T (2024). NutriMine - a serious game modification for Minecraft to support people keeping a healthy diet. Proceedings of the 12th International Conference on Serious Games and Applications for Health.

[40] Aigner C, Eder M, Baranyi R, Grechenig T (2020). recoverApp - a mobile health solution to support people in stationary rehabilitation. Proceedings of the 8th International Conference on Serious Games and Applications for Health.

[41] (2025). Obesity and overweight. World Health Organization.

[42] (2023). DiGA-Leitfaden (stand: 28.12.2023, version 3.5). Bundesinstitut für Arzneimittel und Medizinprodukte.

[43] Hendolin M, European Observatory on Health Systems and Policies (2022). Towards the European health data space: from diversity to a common framework. Eurohealth.

[44] European Health Data Space Regulation (EHDS). European Commission.

[45] Bradner S BCP 14 RFC 2119: key words for use in RFCs to Indicate Requirement Levels. RFC Editor.

[46] Baranyi R (2023). DeapSea: workflow-supported serious game design for stroke rehabilitation. Int J Comput Games Technol.

[47] SNOMED International homepage. SNOMED International.

[48] LOINC homepage. LOINC.

[49] FHIR documents. HL7 FHIR.

[50] IT Infrastructure (ITI) domain. Integrating the Healthcare Enterprise.

[51] 10 cross-enterprise document sharing (XDS.b). Integrating the Healthcare Enterprise.

[52] Kramer M, Moesel C (2021). Tutorial: create an implementation guide with FHIR shorthand. HL7 FHIR.

[53] FHIR Shorthand. HL7 FHIR.

[54] FHIR / sushi. GitHub.

[55] FHIR tools - Visual Studio Marketplace. Visual Studio Marketplace.

[56] HL7 FHIR Shorthand. Visual Studio Marketplace.

[57] HL7 Balloting. Confluence.

[58] Reichardt A (2021). Serious Games: spielen um leben und tod. Deutsches Ärzteblatt.

[59] OpenAPI Initiative homepage. OpenAPI Initiative.

[60] Unity homepage. Unity.

[61] Consolidated text: Regulation (EU) 2016/679 of the European Parliament and of the Council of 27 April 2016 on the protection of natural persons with regard to the processing of personal data and on the free movement of such data, and repealing Directive 95/46/EC (General Data Protection Regulation) (Text with EEA relevance). European Union.

[62] Li Y, Wang H, Yerebakan HZ, Shinagawa Y, Luo Y (2024). FHIR-GPT enhances health interoperability with large language models. NEJM AI.

[63] Implementation guide for NCD-prevention games and apps. Fast Healthcare Interoperability Resources.

